# Development of an open technology sensor suite for assisted living: a student-led research project

**DOI:** 10.1098/rsfs.2016.0018

**Published:** 2016-08-06

**Authors:** James D. Manton, Josephine A. E. Hughes, Oliver Bonner, Omar A. Amjad, Philip Mair, Isabella Miele, Tiesheng Wang, Vitaly Levdik, Richard D. Hall, Géraldine Baekelandt, Fernando da Cruz Vasconcellos, Oliver Hadeler, Tanya Hutter, Clemens F. Kaminski

**Affiliations:** 1 EPSRC Centre for Doctoral Training in Sensor Technologies and Applications, University of Cambridge, Cambridge, UK; 2 Department of Chemical Engineering and Biotechnology, University of Cambridge, Cambridge, UK; 3 Department of Engineering, University of Cambridge, Cambridge, UK; 4 Cavendish Laboratory, University of Cambridge, Cambridge, UK; 5 Department of Biochemistry, University of Cambridge, Cambridge, UK; 6 Department of Materials Science and Metallurgy, University of Cambridge, Cambridge, UK; 7 Department of Chemistry, University of Cambridge, Cambridge, UK

**Keywords:** assisted living technology, wireless sensor networks, open technology

## Abstract

Many countries have a rapidly ageing population, placing strain on health services and creating a growing market for assistive technology for older people. We have, through a student-led, 12-week project for 10 students from a variety of science and engineering backgrounds, developed an integrated sensor system to enable older people, or those at risk, to live independently in their own homes for longer, while providing reassurance for their family and carers. We provide details on the design procedure and performance of our sensor system and the management and execution of a short-term, student-led research project. Detailed information on the design and use of our devices, including a door sensor, power monitor, fall detector, general in-house sensor unit and easy-to-use location-aware communications device, is given, with our open designs being contrasted with closed proprietary systems. A case study is presented for the use of our devices in a real-world context, along with a comparison with commercially available systems. We discuss how the system could lead to improvements in the quality of life of older users and increase the effectiveness of their associated care network. We reflect on how recent developments in open source technology and rapid prototyping increase the scope and potential for the development of powerful sensor systems and, finally, conclude with a student perspective on this team effort and highlight learning outcomes, arguing that open technologies will revolutionize the way in which technology will be deployed in academic research in the future.

## 1. Introduction

With an ever-growing elderly population and increasing financial pressure on health services, assisted living technologies (ALTs) are on the brink of transforming from a niche market to a huge industry [1]. It is preferable from both the patient's perspective and the care provider's to keep people at home for as long as possible, out of the extremely costly residential care system. This project identifies and fills a niche in the ALT market for an integrated sensor platform that allows individuals to retain their independence and dignity for longer than is otherwise possible.

As an example of the increasing challenge of caring for an ageing population, consider the UK. There are currently 3 million people aged 80 or over, with the number of people aged over 85 set to double in the next 20 years, and triple in the next 30 years [2]. This substantial increase will inevitably put an even greater burden on an already under-resourced National Health Service as well as the local authorities that provide residential care. Thirty billion GBP of investment has led to an increase in capacity of around 350 000 beds for the care of older people, meaning that each bed cost an average of around 86 000 GBP [3]. As such, there is considerable scope for technological developments to reduce the number of beds required, even if the initial costs of such a system are high.

For many older people, living with younger family members is not an option, and so they and their families have to make the life-changing decision on when to move into residential care from their own homes. As it stands, either the person lives at home on their own and risks not being found for hours after a serious medical episode, such as a heart attack or stroke, or they move into a care home at a huge cost (typically 500–600 GBP per week [4]) and lose a large portion of their independence. This project sets out to develop a means whereby a compromise can be reached—i.e. the person can remain living at home but with discreet and reliable monitoring systems providing alerts if an incident occurs. Such a system gives the older persons the dignity and security to live in their own homes while keeping them as safe as possible, providing peace of mind for their family.

Existing systems in this market are mostly antiquated and lack the finesse and integration that modern technology allows. In addition, these systems are monolithic and offer no integration with products outside the company's own range. An Innovate UK report [5] published in 2013 identified this lack of interoperability as one of the technological reasons why uptake of ALT was so poor.

Current commercial offerings operate under a closed system, whereby devices purchased from one manufacturer cannot be used with those from another. Social workers who install such systems say that this is extremely frustrating as, while one company may excel in one product area, they often are lacking in others. An open, standardized platform would allow individuals to make choices about which individual devices work best for them, increasing competition, and therefore improving standards across the sector. Table 1 outlines the main features of a number of commercial systems, alongside the system described here, and clearly shows that our open approach compares favourably with other available systems. In particular, none of the current commercial offerings provide the breadth of measurements and analysis present in the described system. Furthermore, the open design means that it is comparably easy to add a feature developed by others for other systems, but not currently present in our own, unlike all the commercial systems detailed. While this may not be directly relevant for end-users, the open nature of the system may be a significant draw for other companies to develop complementary products that provide additional sensing capability within our framework. A survey, carried out in the early stages of the project, showed that there was considerable desire for an assisted living system from older people, that older people thought current offerings were lacking in features and that they were disappointed by the lack of interoperability between devices of different manufacturers. The benefits of open source technology in a ‘smart home setting’ were furthermore discussed in a recent paper [10].

**Table 1.** Commercial assisted living technology solutions compared with the system described here. Note that no one commercial system has all the features of another and only Telehealth Sensors, which provide individual devices rather than a complete platform, are interoperable.

system	interoperable	motion sensor	door sensor	bed sensor	power monitor	fall detection	activity analysis	cost	max. no. devices	data presentation	alerts	ref.
Canary	X	√	√	X	X	X	√	18 GBP per month and 348 GBP upfront	8	individual events over time	email and SMS	[6]
JustChecking	X	√	√	X	X	X	X	30 GBP per month and 590 GBP upfront	10	individual events over time	SMS	[7]
Telehealth Sensors	√	X	√	√	X	X	X	—	unlimited	—	—	[8]
Alarm.com Wellness	X	√	√	√	X	X	√	around 100 USD per sensor	unlimited	individual events over time	email and SMS	[9]
this system	√	√	√	X	√	√	√	—	unlimited	individual events over time plus data analysis results, e.g. activity-level indications	none	—

A number of companies, such as Microsoft and Google, have developed online health record systems where a medical history of an individual is stored online and can be accessed by healthcare professionals. Microsoft's HealthVault product allows health and fitness data collected from devices such as pulse rate monitors to be logged online and can provide a more fine-grained medical history than the traditional system of doctor-curated records. However, a number of such systems, such as Google Health, have ceased to exist, with users being forced to export their data to competitors' systems or, in some cases, being left without access to their data at all. The open design approach taken in this project prevents such migration problems and enables different complementary analyses and sensing measurements from different providers.

Current systems provide a utilitarian approach to home monitoring, i.e. they provide the crucial elements required to monitor older people in their homes and nothing more. An integrated, ‘always-on’ sensing system may be perceived as invasive and Orwellian, particularly if the older person recognizes no benefit from the system [11]. A social application for Android tablets that allows family members to share photographs and messages simply and easily with their elderly relatives has already been developed in a separate context. Having such a social interaction feature built into an ALT set-up would help counter this, and engage the older person with the technology rather than alienating them [12].

Having identified these avenues for ALT system development, a postgraduate cohort comprising the full 10-student 2014 intake of the Engineering and Physical Sciences Research Council (EPSRC) Centre for Doctoral Training in Sensor Technologies and Applications (Sensor CDT) developed a new, open and easily extendible ALT system in 12 weeks between April and August 2015. As the Sensor CDT is, by design, highly interdisciplinary, each student came from a different background, both culturally and in terms of the initial skill sets possessed, ranging from biochemists to engineers, physicists, chemists and materials scientists. This diversity was a valuable asset for the successful completion of the project in the short time available.

The general topic of ‘developing assisted living technologies for older people’ was given to the cohort by Sensor CDT staff, but the remaining details of the project were entirely conceived, planned, directed and executed by the students themselves. The work was shared between the students, so that each member of the team could contribute most effectively given their own skill set and research interests.

The project comprised two phases: in the first phase (May–June 2015), proof-of-principle prototypes were developed and a first integration of the system was attempted, whereas the second phase (June–August 2015) was dedicated to achieving a fully functional and integrated ALT system. The project was initially subdivided into six technical workpackages and one administrative workpackage for each of the two stages. The modular nature of the system allowed the immediate assignation of some workpackages to individual members of the team already possessing the necessary skills and knowledge for their successful completion. Other workpackages would require any individual member of the cohort to research and develop new skills, and so these workpackages were shared between multiple members of the cohort. In addition to honing research and technical skills in preparation for the later PhD project phase of the students, the project also provided a valuable platform on which to develop valuable team skills, an aspect not usually covered in traditional, single-discipline and individually performed PhD programmes.

This article begins with an overview of the system produced and the general methodologies used during its development, including project management. Each individual hardware component of the system is then detailed, along with a description of the software developed to link the devices together and analyse the data collected. We conclude with a comparison of our developed system, highlighting its advantages over current commercial systems, and provide an outlook on potential future developments within the ALT sector. In addition, we reflect on the experience of performing team-led research in an academic setting and summarize our key findings in terms of using open technologies to develop effective solutions within comparatively short time frames.

## 2. Project definition and methodology

### 2.1. System overview

The system developed uses wireless sensors placed at various locations around the house, which indirectly monitor the older person's wellbeing. The aim is to perform this non-invasively and thus to create a solution which is more easily accepted by the older person than those provided by the direct monitoring of wellbeing through, for example, personal emergency alarms. Table 2 summarizes the indirect indicators of wellbeing which we have chosen to monitor, along with the non-invasive sensors used.

**Table 2.** Examples of different home-based measurements, the sensors used to measure them, what can be inferred from the measurements, and the advantages of the particular sensors chosen.

measurement	indication	sensor	reasons for choice of sensor
electricity usage	— indication of device usage (e.g. television or kettle) — energy usage in the house	— hall effect sensor: measures instantaneous power consumption	— does not require direct interaction with the high-voltage mains electricity supply — fast response time — not affected by ambient conditions
movement around the house	— older person awake and moving around the house — lack of movement when movement is expected — movement in particular rooms (e.g. excessive use of toilet)	— passive infrared sensor: detects changes in infrared levels caused by, for example, a moving human body	— low cost — binary output — one sensor can cover an entire room
changes in light levels	— lights being switched on or curtains opened — movement around the house	— light-dependent resistor: produces a voltage proportional to light level	— low cost — easy to use, few components required
noise levels	— movement around the house	— microphone	— low cost — highly sensitive — wide frequency sensitivity
room temperature	— heating systems are being used adequately — appropriate room temperature	— thermistor: produces a voltage proportional to temperature	— low cost — no additional components required
door openings/closings	— family members entering or leaving — older person leaving or returning — non-returns	— reed switch: binary output for door open/closed state	— binary output directly corresponds to state of door — small physical size
falls	— activity levels — accidents	— accelerometer: measures acceleration in a given axis — barometer: measures pressure, which indicates altitude when temperature compensated	— small size — low cost — high accuracy for fall detection

Over time, the data from these wireless sensors allow a picture of a person's daily habits and routine to be developed. Deviations from daily patterns can be used as indicators of a potential crisis, such as an incapacitating accident, or an onset of chronic disease.

An integrated solution was developed, comprising two key parts: an on-person device (OPD) for use outside the house, providing a simple and easy-to-use messaging system and a suite of fully integrated and complementary sensors, and an in-house system of wireless sensors.

The OPD system is for situations when the user is away from home. The incorporated messaging system permits two-way phone calls to be made to a predefined telephone number upon pressing a single dedicated call button. A second button allows the user to send the current GPS location via an SMS message to a different, or the same, predefined telephone number. This provides a convenient, easy-to-use interface for the older user, allowing them to make contact easily to, for example, arrange pick-up from a shopping trip. Additionally, in an emergency, this can be used to make contact with a relative and provide location information quickly and easily.

**Figure 1.**
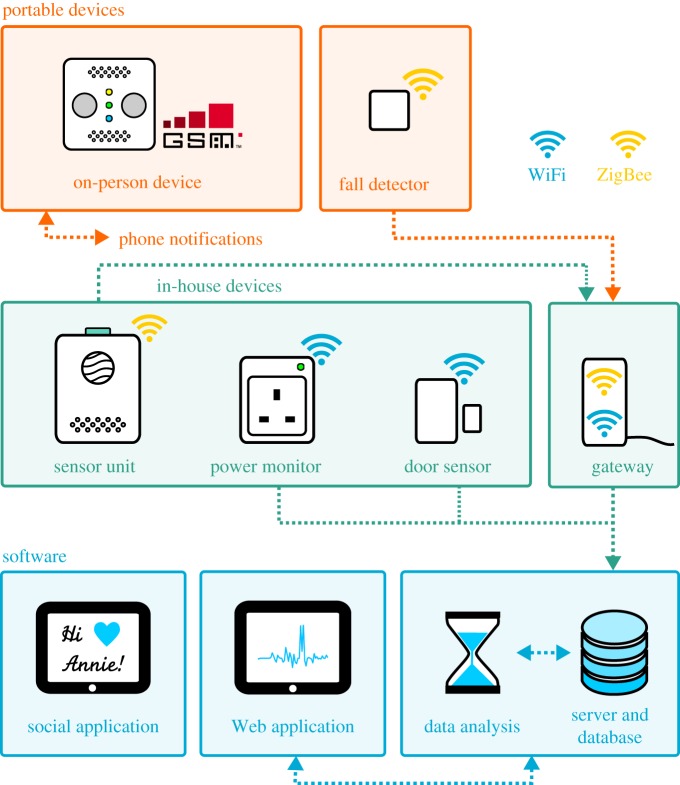
Diagrammatic overview of all components of our modular system. Portable devices are carried by the person and communicate either over GSM to a predefined contact, for the OPD, or over ZigBee to our gateway and associated online infrastructure. The in-house devices all communicate their measurements to the online infrastructure, either directly via WiFi or through the gateway via ZigBee. Our online infrastructure records measurements from all the devices to a database, and provides an interface for accessing measurements for the purpose of data analysis and end-user display. Both raw measurements and analyses can be viewed by a user using our Web application, with a social application facilitating communication between the older person and their family.

The in-house system consists of a number of wireless devices that collectively detect presence and activity within the house, allowing family members or carers to remotely check on the safety and wellbeing of the older person with minimal invasion of privacy. The system includes door sensors for entry/egress monitoring, power monitors to measure device usage and power consumption, and a multi-functional sensor unit, including passive infrared movement and noise-level sensors. In addition to these domestic environment sensors, a wearable fall detector was developed. This is a small, low-cost, low-power consumption device used to monitor personal activity levels and detect falls. It was designed to be worn unobtrusively on the person as a brooch, belt buckle or in a pocket. A diagrammatic summary of these devices is given in figure 1.

All in-house devices are connected to a dedicated online infrastructure, which collects data from the individual devices, stores historical measurements, and makes raw data and analyses available to authorized users (e.g. family) through an easy-to-use Web interface. As well as providing an overview of the current state of the monitored individual, this may also be used to detect long-term deterioration in health and wellbeing. To transfer data from each device to the Internet, the Particle.io platform was chosen, which allows a microcontroller to send data over a WiFi connection. Full details of the exact hardware used, beyond that presented in the Methods and discussion section, are given in the electronic supplementary material. Data from each device are sent to the Particle.io servers where they are rebroadcast and saved by listening code into a database. This database is then used to present data online and can also be used for long-term analyses, such as training machine learning algorithms to establish, and detect deviations from, daily routines.

### 2.2. Open technology

While open source software has existed since at least 1983, with Richard Stallman's publication of the GNU Manifesto and launch of the GNU Project, it is only recently that it has become cost-effective and easy to interface software with custom-purpose hardware, through efforts such as the Arduino and Raspberry Pi projects [13,14]. Furthermore, the recent explosion of interest in the ‘Internet of things' has caused a large number of manufacturers to produce Internet-connected devices and services with easy-to-use interfaces and application programming interfaces (APIs). In addition, efforts such as Codecademy and Coursera have lowered the barrier to entry for open source software development, whereas social development platforms such as GitHub and Instructables have encouraged individuals to share their software and hardware projects online, making it easy for everyone to develop new technologies and devices by combining and modifying projects documented on the Web [15–18]. In research, too, open source technology is rapidly generating impact, permitting researchers to implement sophisticated instrumentation at low cost [19–21].

We decided to fully leverage these developments to allow us to focus on the novel aspects of our technology rather than spending development time re-implementing, for example, wireless communications and low-level hardware control. This allowed us to move rapidly from concept to fully working prototypes, which took just 12 weeks—an inconceivable goal just a few years ago. In keeping with the spirit of open technology, our outputs are also fully open, with software code and hardware designs made freely available online (see the electronic supplementary material). We have developed a truly open and extensible system that can be easily developed further or repurposed by anyone with minimal developmental overheads.

### 2.3. Rapid prototyping

Owing to the short time scales of the project, it was necessary to produce software and hardware prototypes rapidly to check their function and guide future development. By using an iterative development cycle, key functionalities were quickly developed, tested and refined, and the final product comprised the results of many iterations on individual features. This was only possible owing to developments, within the past few years, that have made three-dimensional printing, printed circuit board (PCB) production and electronics prototyping cost-efficient, easy-to-learn and readily available.

#### 2.3.1. Three-dimensional printing

Three-dimensional printing technology was used to generate prototypes for housings and casings for the hardware developed. The ergonomics and design of the prototypes were key considerations, as usability is a key requirement for devices for older people owing to their potentially limited sight or dexterity. Commercial CAD software (Autodesk Inventor) was used to design the models on a computer, after which they were printed in ABS plastic using a commercial three-dimensional printer (MakerBot Replicator 2X). Three-dimensional printing is a highly flexible prototyping method and allowed many iterations of designs to be printed and tested before the final design was produced. In addition, the low material usage and cost per print meant that prototyping was limited not by our ability to manufacture components, but by our ability to design them.

#### 2.3.2. Electronic hardware

The development of the technology in the short time scales available was enabled by the availability of low-cost electronics prototyping boards such as the Arduinos, Raspberry Pis, Particle Photons and Freescale mbed development boards used [13,14,22,23]. This allowed rapid development of early prototypes and associated software. The design methodology for the electronics development was first to test circuits or sensors using breadboards, in conjunction with the development boards, with transferral to more permanent stripboard afterwards. Once the design was finalized it was possible to produce custom PCBs for the circuits, either still using the development boards (as was done for the power monitor, door sensor and sensor unit) or using a microcontroller system-on-chip directly on the PCB (as was done with the fall detector).

A number of prototyping boards were used to develop the system, all based on the ARM architecture. The mbed platform was used to develop early-stage prototype devices. This platform, provided by ARM, includes low-cost development boards and surrounding real-time operating system, cloud services and integrated development environment, which collectively allow for rapid development and deployment. The particular development board used (mbed FRDM KL25Z) includes an ARM M0-Cortex microprocessor and features a number of interfaces for easy communication to and from other integrated circuits.

To develop WiFi-enabled prototypes, we chose to use the Particle Photon development boards from Particle.io. These contain a powerful ARM Cortex-M3 microprocessor and a Broadcom WiFi chip. Low-level firmware is preloaded onto the device by the manufacturer, which enables and configures the WiFi communications and allows higher level C++ code to be written to control the device. In addition to the hardware, a cloud platform for the logging of events and messages, and code development, is also provided. This provides a secure connection to interact with the Particle Photon through APIs, allowing the development of our associated online infrastructure.

#### 2.3.3. Project management and structure

Detailed project planning and management were implemented to ensure the successful completion of this research challenge as a group of 10 students. The project split into two separate phases. In the first, proof-of-principle devices were produced, whereas the second focused on developing and testing fully functional devices. The modular nature of our system suggested that the work could be best divided by creating workpackages for each device, along with two more for the online infrastructure development and data analysis software. As many device features are interdependent, we produced Gantt charts to guide the development timeline and ensure that dependencies between workpackages were satisfied within reasonable time scales, minimizing downtime.

Key feature sets and production stages were identified as milestones, with the project not being allowed to transfer to the second phase without all workpackages meeting the milestones of phase 1. To further ensure accountability and to keep the project on track, three students were selected as project leads: one as a lead in charge of the technological development of the devices, one to lead all administrative tasks such as financial accounting and logistics, and one as a project manager with overall responsibility for the project and with the final say on design decisions.

#### 2.3.4. Mentoring sessions

Throughout the project, weekly mentoring sessions with a number of experts in the fields of medicine, social care, telecoms and medical start-ups gave vital feedback on both the implementation of our designs and the needs and requirements of the users. This was particularly useful when developing the initial proposal for the system and ensured that we were developing sensor technology that was beneficial, usable and feasible to develop.

Understanding the needs and requirements of the target user was key. We were invited to visit a ‘Smart Flat’ equipped with the latest ALTs. This exposed us to technology already available on the market and installed in a typical set-up. This also provided insights into current limitations or challenges associated with ALT, and design criteria to consider to ensure engagement and uptake with older users. Engaging with specialist social workers who provide ALT systems was invaluable as it gave a real understanding of the daily challenges faced by older people and how technology can be used to increase quality of life.

No direct technical guidance was given at any stage of the project, beyond suggestions that the devices should have long battery lives, etc. This meant that we were free to select technologies based on a balance of their technical merits, cost and open source nature. We do not suggest that our selections are necessarily the best for a potential mass-produced, commercialized version of our system.

## 3. Methods and discussion

In this section, we describe the devices and software developed in general terms, with full details of the hardware used provided in the electronic supplementary material.

### 3.1. Power monitor

Monitoring the power consumption of a frequently used device, such as a kettle or television, provides a non-invasive, basic measure of the activity of an individual. Our power monitor takes its inspiration from the Japanese-developed iPot, which monitors the tea-making habits of the user [24]. As tea-making is often a regular activity, an alert can be raised if tea is not made in a given time period. Our device generalizes this concept by inserting a power monitor between the wall socket and an appliance. The appliance can be anything which is used regularly such as the aforementioned kettle, or a television, radio or toaster. Passive monitoring of these appliances and subsequent data feeding into our monitoring algorithms can help to build a picture of the activity of the older person.

The functional requirements of the power monitor were twofold: to detect whether the electrical device is on or off, and to measure the energy being consumed. The former of these requirements is a simplification of the latter, in that the device is turned on when it is consuming power (i.e. the power reading is non-zero). For the sensor inside the power monitor device, a Hall effect sensor was chosen for its small size (Allegro ACS723 and ACS717 integrated circuits), and for the galvanic isolation that is offered by this type of sensor. Hall effect sensors are commonly used as proximity sensors for magnetic objects in, for instance, a security system to detect a window being opened. In this application, however, the sensor is placed close to the current-carrying wire of the appliance, so that induction is maximized, and the output is a direct measure of the current drawn.

In order to ensure that a full range of appliances, from low-power radios to high-power kettles, could be monitored, two Hall effect sensors were used—one sensitive to a high current range and the other to a low current range. This provided the wide dynamic range required for generalized application.

**Figure 2.**
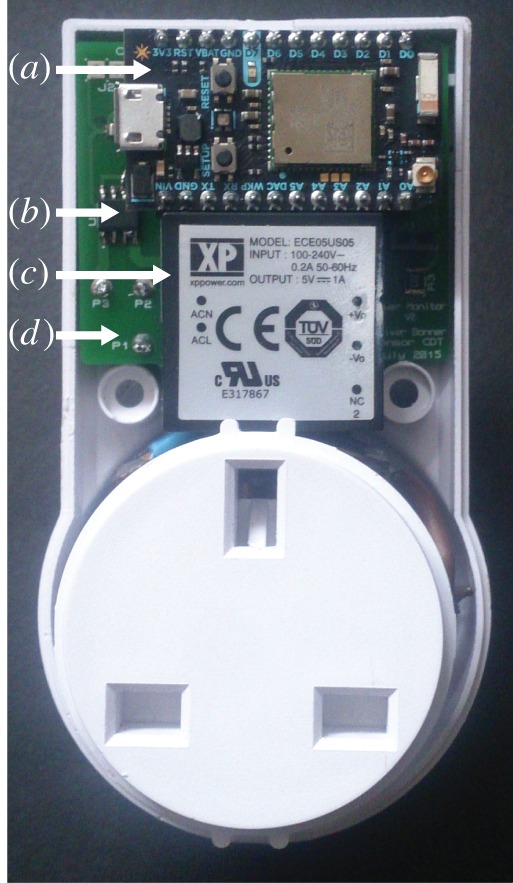
The internal configuration of the final design of the power monitor. The white plastic housing is taken from a commercial wireless power-switching device. (*a*) The Particle Photon microcontroller and WiFi development board. (*b*) One of two Hall effect integrated circuits for measuring current flow. (*c*) Switch mode power supply, connected to the same mains supply as the device being monitored and powering the power monitor. (*d*) Custom printed circuit board to hold and connect all components used.

**Figure 3.**
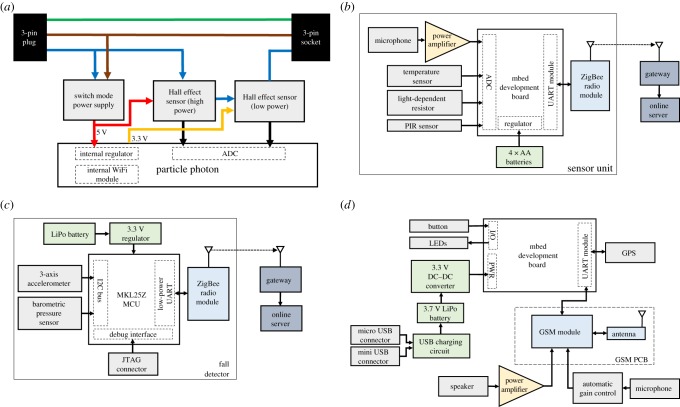
Block diagrams of devices produced. (*a*) The power monitor, which draws power from the three-pin plug and measures the current drawn by a device, via two Hall effect sensors, through the three-pin socket. (*b*) The sensor unit, powered by AA batteries and which measures the temperature, light levels, noise and motion (via the passive infrared (PIR) sensor) and communicates wirelessly with the gateway, which in turn relays measurements to our online infrastructure. (*c*) The fall detector, a battery-powered wireless device which uses a three-axis accelerometer and barometric pressure sensor to detect falls through a decision tree algorithm. (*d*) The on-person device, a wireless battery-powered communications device with simplified interface. Location information, provided by a global positioning system (GPS) module, can be sent via SMS message to a predefined contact, with two-way telephone calls to the same, or other, predefined contact being facilitated through our GSM printed circuit board, microphone and speaker. To provide the necessary current for initiating a call, a 3.3 V DC–DC converter in a ‘boost’ circuit is used.

A rapid prototyping WiFi-enabled board, the Particle Photon, was used to measure the output from the Hall effect sensor and communicate the on/off state of the appliance to the central processing hub. A low-profile switched-mode power supply (XP Power ECE05US05) was used in parallel with the appliance to power the sensing electronics. A plastic case from a wireless power switch product was used to enclose the device, to produce an electrically insulated and safe casing. A PCB was designed and produced to fit within this casing. The internals of the final device can be seen in figure 2. A block diagram of the final design produced is presented in figure 3*a*.

### 3.2. Door sensor

During the early consultation phase of the project, an assisted living specialist suggested that the inclusion of a door sensor would allow the home activity to be interpreted more easily. When individuals enter or leave the house, this event can be logged and help in the interpretation of the data from other sensors.

The functional requirements of the door sensor are to detect state changes in the door, and to operate for a period of months without the need to change the battery. The Particle Photon, as used in the power monitor, incorporates a deep sleep mode with wake-up being triggered on an external interrupt signal. This allowed the simple development of code that met the device specification.

For the sensor itself, a simple magnetically actuated reed switch was chosen. This type of sensor is much lower cost than other sensor types such as Hall effect proximity sensors. The sensor was placed in series with a pull-down resistor with the midpoint connected to a digital input on the Photon. In the developed software running on the Photon, the device was set to wake up from deep sleep mode when the state of the digital pin changed; the state of the door can then be sent over the integrated WiFi connection to the Internet server.

We chose lithium thionyl chloride battery technology (Saft LS 14500 W) for its excellent power density and stable voltage profile [25]. The estimated battery life, given heavy daily usage, was more than eight months. The cost to produce the door sensor in volume with listed components is approximately 12 GBP. Fifty per cent of this cost is associated with the Particle Photon board, and significant savings would be made if a custom-designed wireless platform was used. The use of available open source technologies, as done here, demonstrates the enormous potential, however, to prototype sophisticated devices in very short time frames, here less than 12 weeks.

### 3.3. Sensor unit

The key purpose of the sensor unit is to monitor movement within a room unobtrusively, so that any abnormalities in activity can be detected. To perform this function, it is equipped with a suite of sensors that indirectly measure indications of activity levels. Local analysis is performed on the sensor readings, after which the results are transmitted wirelessly to a central server via the gateway (see §3.5), allowing it to be displayed on the Web application (see §3.8). The low cost of the device, approximately 75 GBP for a prototype, enables multiple devices to be installed throughout the home in key rooms, with the estimated battery life of one month ensuring only infrequent maintenance is required.

An mbed development board was used as the central processor, reading measurements from the sensors and communicating with the wireless module to transmit the data to the online infrastructure. This platform is low cost and low power (20 mA current consumption) and has an on-board 16-bit ADC, sufficient for a wide range of sensor inputs. Four sensors were integrated into the unit: a passive infrared sensor to detect the movement of warm bodies, a temperature sensor, a light-dependent resistor to detect ambient light levels, and a microphone to detect noise caused by movement.

The initial prototype developed was powered by the mains supply as this meant considerations concerning power consumption were unnecessary. However, after consultation with healthcare professionals, it was developed into a battery-powered device to provide greater flexibility and versatility in locating units throughout the home. Changing batteries in the device could be difficult for older persons, particularly if sensor units are located in difficult-to-access locations. We thus aimed to minimize power consumption and produce a long-lifetime device. A number of different wireless technologies were investigated for their suitability in this low-power scenario [26]. Despite having sufficient range, the power consumption of WiFi was too high to achieve a reasonable battery life. Conversely, low power consumption could be achieved with Bluetooth or Bluetooth low energy, but the range, being only a few metres, would be insufficient to cover a house. We selected ZigBee, as it provides a good balance of low power consumption and suitable range, with its simple design allowing rapid prototyping of a wireless device. A block diagram of the final sensor unit design produced is presented in figure 3*b*.

The software for the device was developed using the mbed C++ IDE. This polled the sensors at 10 Hz, calculating the measurement average and standard deviation over a 5 min window, with the results being transmitted to the gateway once every 5 min. This approach of using on-board processing and a low rate of transmission significantly reduced the power consumption of the device; transmitting the raw data at 10 Hz would require much more power, acting against our goal of a long-lifetime device.

A real-world test of our sensor unit, along with our power monitor and door sensor devices, feeding data into the data analysis pipeline and online Web interface, is described in §3.7.

### 3.4. Fall detector

A fall detector device was created in order to remotely detect falls within the user's home, by measuring changes in acceleration and tilt on the person using a triaxial accelerometer, coupled with a barometer. To minimize the physical size and power consumption of the device, a PCB with a Freescale KL25Z microcontroller, additional passive components and a 3.3 V regulator circuit was developed. The barometer and accelerometer were both connected to the inter-integrated circuit bus on the microcontroller as this allowed for simple polling of the sensors from the same bus. A lithium polymer (LiPo) battery was used to power the device and there was a connection to a ZigBee module over universal asynchronous receiver/transmitter (UART) for wireless transmission. This allowed for a small, unobtrusive fall-detection device with an estimated battery life of one week.

In our initial testing of fall detection, a combination of dynamic time warping [27], to allow a measurement of the similarity between time series, along with machine learning classification algorithms were used to determine whether or not a fall had occurred based on real-time data transmitted to a computer over a USB connection. However, transmitting data wirelessly over ZigBee at the rate required to accurately sense falls would be extremely energetically costly, and either lead to a very short battery life or require a much larger device with more batteries. Additionally, the microcontroller was found to have insufficient RAM to house a large collected training dataset, making on-the-fly learning impossible. Therefore, in the final version of the device, a non-machine learning algorithm was implemented on the device's microcontroller. This final algorithm was an adapted version of the approach used by Tolkiehn *et al*. [28] where a series of thresholds were required to be exceeded in order for a fall to be detected. Preliminary testing gave results with accuracy, sensitivity and specificity of fall detection greater than 85%. The algorithm was implemented in C such that it could be downloaded onto the microcontroller using the Kiel *μ* Vision IDE. Additionally, using the scikit-learn library in Python, a decision tree algorithm was used to find appropriate thresholds for the algorithm developed here, based on real-time data transmitted over a USB connection. A block diagram of the final fall detector design produced is presented in figure 3*c*.

### 3.5. On-person device

The OPD is a hand-held mobile communication device that allows the user to easily communicate using the global system for mobile communications (GSM) network to make calls and send SMS messages when out of reach of the home-based system. It has been designed to be small, portable and easy to use and includes a global positioning system (GPS) module for location awareness. The device has only two buttons, one to send an SMS message to a predetermined telephone number containing the current GPS location and the second to make a phone call to the same, or different, predetermined telephone number.

A simplified block diagram of the final version of the OPD is shown in figure 3*d*. At the heart of the device is a KLZ25 mbed development board, which communicates over a UART bus to the GSM and GPS devices. A full GSM PCB has been developed using a dual-band Gl865-DUAL Telit GSM surface mount chip that communicates with the microcontroller using attention (AT) commands over UART. The PCB design includes all the requisite data lines, power lines and level shifters, and connects to a microphone and speaker. The GSM chip can require up to 1.5 A of current for a short period of time when initiating a call, despite requiring a much lower current (30 mA) during normal use. To provide sufficient current during peak demand, a 2500 mAh, 3.7 V LiPo battery was used in conjunction with a single-ended primary inductive SEPIC DC–DC converter (Linear Technology LTC1872). The device can be recharged via a USB connection or using an inductive pad. We estimate the battery life of the device at six weeks, assuming the device is used for 2 h per day.

### 3.6. Gateway

While the power monitor and door sensor communicate directly with the Particle system over WiFi, the fall detector and sensor unit do not, as a WiFi connection on these devices would consume too much power. Hence, a lower power radio protocol was used to communicate readings from these devices to a mains-powered gateway, from which these readings were relayed to the Particle system over WiFi. The gateway hardware is very simple, consisting solely of a Particle Photon microcontroller and a ZigBee wireless module.

As the gateway receives measurements from many devices, but the Particle system only knows about the existence of the gateway, it was necessary to devise a system that includes identifying information in the readings that are broadcast by each device. For simplicity, and ease of later processing, each broadcast was prefixed with a unique, device-specific alphanumeric code. The custom listener code in the online infrastructure (see §3.8) then listened to the Particle event stream for the gateway and used the unique identifier to ‘fix’ the identity of the device before adding the measurements to the database.

### 3.7. Data analysis

**Figure 4.**
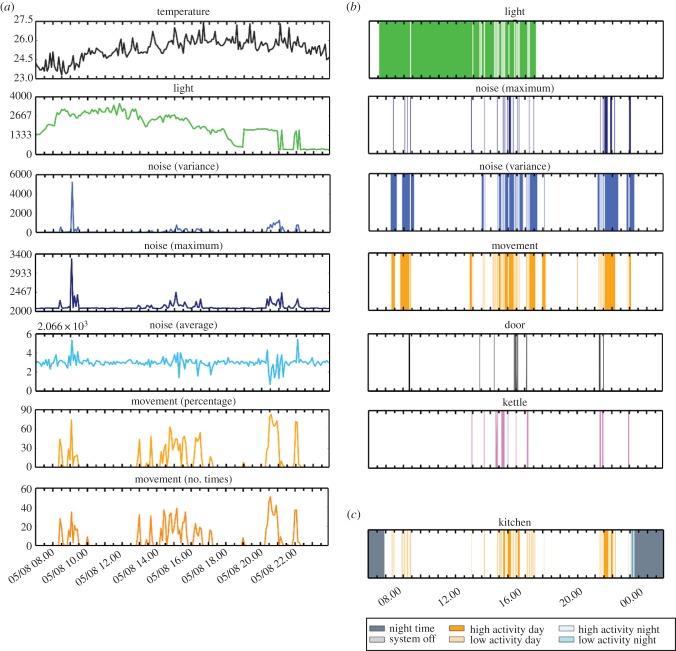
Demonstration of how raw sensor readings from the sensor unit, power monitor and door sensor can be used to produce a single plot with an indication of activity levels throughout the day. (*a*) Sensor readings transmitted from the sensor unit to the gateway. (*b*) Binary sensor outputs produced by thresholding sensor readings and additional binary outputs from the power monitor and door sensors. (*c*) Single plot showing the activity throughout the day determined using a combination of all thresholded, binary sensor results.

All devices in the sensor suite provide simple measurements that, while informative on their own, provide much more information on activity and wellbeing if combined and analysed as a whole. This section details the analysis pipeline and applications to a real-world testing scenario, in which two sensor units were installed in a home environment, along with power monitors and door sensors. The combined data from these devices allowed a determination of room-specific activity levels. Diurnal patterns were easily detected, along with movement around the house. A typical output is shown in figure 4 (see also electronic supplementary material, movie S1).

The analysis algorithms were executed by the server in regular time intervals to sequentially calculate the activity in each room based on new database entries. The results were made accessible to the user via a custom Web interface. To achieve this, four goals had to be met: the incoming data had to be pre-processed in order to be comparable, the information from the different devices had to be combined, the degree of activity had to be calculated and, finally, the activity had to be formatted in an intuitive manner.

For example, the activity in a kitchen was calculated. In this kitchen were one sensor unit, one power monitor connected to an electric kettle and one door sensor fitted to the back door of the house. The data from the sensor box were loaded first, which provided information about the average light, noise and movement levels within 5 min intervals. The data of each measurand were smoothed over time using a Gaussian filter (with a standard deviation of 5 min), normalized, and then thresholded with a measurement-specific threshold. The thresholding was used to achieve a binary high/low interpretation for each parameter. Hence, the direct comparison of different physical measurements was possible—the more measurands were identified above certain thresholds at given point in time, the higher the overall activity level.

After having analysed the data from the sensor unit, the data from the other two devices were loaded. The door sensor transmitted a single 1 when the door was opened, and a single 0 when it was closed. The power monitor transmitted 1 continuously (at 1 Hz) while an appliance was in use and a single 0 to signal it turning off. Mapping of these data to the other continuous measurements of the sensor unit allowed contextual interpretation of the data: a door opening and closing event followed by low activity in all rooms would indicate the older person had left the house. Note that, owing to how the system is designed and the data represented, it is deliberately impossible to identify the person and the exact nature of their activity. This protects the privacy of the monitored individual, while allowing the detection of an unexpected rise or drop in activity.

### 3.8. Online infrastructure

By using the Particle.io platform [22], event messages detailing power cycling and user-defined events processed by the Particle Photon microcontroller used in our devices are provided only as a stream (as JSON objects via an HTTP-accessible API endpoint). While events are broadcast to stream subscribers in near real time, the service does not provide any means to log events, and so historical event details are not available. As such, we produced a Python program to listen to events from all our devices and save the details to an SQL database. This was run using the nginx Web server, through a Web server gateway interface, on a Linode virtual private server. Historical data were then made available in JSON format through a custom Python-powered API running on the same system.

**Figure 5.**
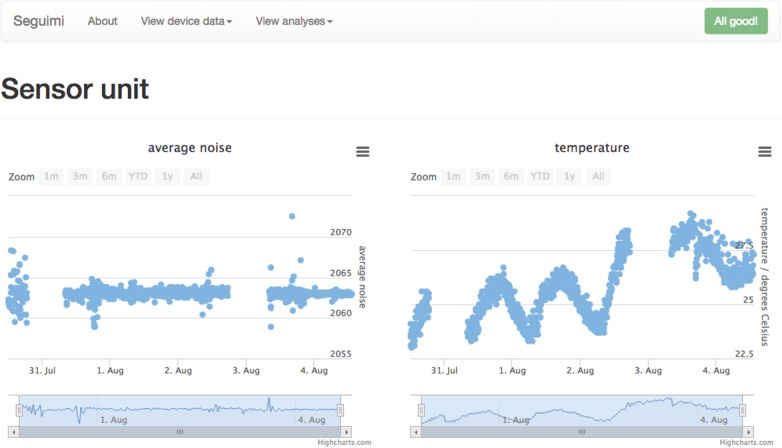
A typical display produced by the online infrastructure, showing the average noise levels and temperatures recorded by a sensor unit over 5 days. A diurnal variation in temperature is clearly visible, with increased noise variance due to a party being shown during the evening of 30 July (left-most data points). Note that our system is robust against connectivity issues, with the system continuing to record data after a disabled Internet connection was reinstated on 31 July and 3 August. Views for different devices, and plots of total analysed activity for different rooms, are accessible through the navigation menu at the top, next to the Seguimi header—the internal name for the sensor suite.

This API is then used to provide data for online graphs of activity, accessible by the user and selected family members, carers, etc. JSON objects are requested from the API and plotted using the Highcharts JavaScript library. Our developed PHP code controls the API requests made and combines the plots into a Web page detailing either a single device or a single room. Figure 5 shows a typical display of our Web interface, with plots representing activity over time in a kitchen.

#### 3.8.1. Tablet computer social interaction application

Many older people have limited social interactions that can affect their quality of life. To improve user uptake, compliance and satisfaction, we augmented the system with an Android application that allows a tablet computer to act as an easy-to-use social interaction platform. The application allows messages, pictures and videos to be securely shared by the user's friends and family to a tablet running the application, located within the user's home. In particular, we chose to use end-to-end encryption to allay privacy concerns, ensuring that messages and photos are not viewable when stored on our server for delivery. The application requires minimal interaction from the older person, but enables family to interact frequently and easily with them. There is scope to use the platform to send reminders to the older relative if required.

## 4. Conclusion

Our project has resulted in the development of a suite of devices that can be used to extend the period of independence of an older person and provide family and carers with a mechanism to monitor the wellbeing of a loved one. Initial results demonstrate that activity levels and daily patterns can be inferred by the system, allowing the user's family to respond to sudden events or changes in behaviour and to investigate accordingly. More subtle changes in behaviour, observed over a longer period, and which may be indicative of gradually decreasing wellbeing, can also be identified, allowing the provision of appropriate support or medical intervention.

An entire ‘starter kit’ of the system, comprising one OPD, two fall detectors, three sensor units, two power monitors, two door sensors and one gateway, has a prototyping cost of only 550 GBP. Considering the effects of the economies of scale, this is comparable to, or lower than, existing and much less comprehensive systems and far lower than the costs associated with living in a care home. The system also has the potential to save national health services significant amounts of money associated with hospital admissions by detecting health conditions earlier, allowing earlier intervention and keeping older persons out of hospital in the longer term. Additionally, the system could be used when older patients are returning home after a period in hospital to ensure that no complications occur, and to allow remote monitoring of their wellbeing.

The system also can relieve the pressure felt by many older people to move into a care home, as their family can be assured of their wellbeing while they are living independently. To maximize user compliance and acceptance, it was important to design a system that was as non-invasive as possible and which retains the privacy of the home-dweller. By only indirectly monitoring the activity of the older person, rather than using cameras or detailed recordings of the movements, their privacy invasion was minimized. This approach was commended by many of the target market individuals and social care providers we consulted, and we were careful to maintain this in the design of the system.

Future development work of the system would be best focused on producing a system that is accepted and actively used by the target group, using the technological framework developed. It has been reported that uptake of ALT systems has been comparatively poor, with the ‘technologically focused’ method of development being criticized for not including input from studies of older people directly [29]. Furthermore, a significant number of older people develop dementia, and this can greatly affect the usability and efficacy of an ALT system [30]. Taking these factors into account when designing an improved version of the system would further distinguish our approach from the current commercial alternatives and should lead to increased uptake and acceptance.

Although extensive further testing is required before a commercial offering is possible, initial tests have demonstrated the benefits of the system, with interest being expressed by potential manufacturers and corporate partners. The end-to-end whole-system approach that we have adopted, along with the integration of data collected from multiple device and sensor types, coupled with the use of open standards to allow new sensor devices to be added in the future, has been noted as a unique and defining strength of the system.

For a student team, the project was also a valuable learning experience. Knowledge of the healthcare sector was gained and, additionally, interdisciplinary technical knowledge and skills were developed. Many team members worked in areas outside their expertise, providing valuable insights into different areas of technology and science. The project allowed for knowledge and skills transfer between individuals in the team—particular highlights include PCB design, surface-mount soldering, software engineering and how to organize and perform research effectively as a team.

As the project was completely student-led, this required significant team organization and project management. The team was required to maintain an accurate record of finances, deal with suppliers and also make project management decisions affecting the direction of the project using feedback from mentors. Working with mentors with backgrounds ranging from business, healthcare, social care and research also provided invaluable experience.

This project has demonstrated that recent developments in open technology and rapid prototyping have vastly increased the research capability to turn ideas into sophisticated sensor technology systems in very short periods of time. The present and similar projects clearly demonstrate the power of such open systems to empower research [31,32]. Collaborating in a cross-disciplinary team, as practised here, proved to be effective in overcoming bottlenecks and in reaching a common research goal on a short time scale. A fully operational sensor suite was conceived, designed, developed and tested by 10 students with minimal research funds, within a 12-week time frame. In addition to a learning experience, useful technology with real potential to compete against commercial solutions has been developed in a project which has the ability to make a significant beneficial impact on society.

## Supplementary Material

Open-source software produced

## Supplementary Material

Original hardware design files

## Supplementary Material

Bill of materials
