# Supplementary material for: Development of an open technology sensor suite for assisted living: a student-led research project
Source: Interface Focus. 2016 Aug 6;6(4):20160018. doi: 10.1098/rsfs.2016.0018 (PMC4918835; doi:10.1098/rsfs.2016.0018)
Supplement: Open-source software produced [file rsfs20160018supp1.pdf]

Software developed

All developed software is open-source and can be downloaded from:

https://github.com/Sensor-CDT-14-15
