# Supplementary material for: Development of an open technology sensor suite for assisted living: a student-led research project
Source: Interface Focus. 2016 Aug 6;6(4):20160018. doi: 10.1098/rsfs.2016.0018 (PMC4918835; doi:10.1098/rsfs.2016.0018)
Supplement: Bill of materials [file rsfs20160018supp3.pdf]

Components used in devices produced

Gateway

- Photon microcontroller (Particle)
- ZigBee wireless communications module XB24-AUI-001 (Digi International)

Sensor Unit

- FRDM-KLZ25 M0+ MCU (NXP Semiconductor)
- ZigBee wireless communications module XB24-AUI-001 (Digi International)
- Temperature sensor AD22103KTZ (Analog Devices)
- MEMS microphone ADMP401 (Analog Devices)
- Passive infra-red movement sensor EKMC1603111 (Philips)
- Light-dependent resistor MSL 4972 (Advanced Photonix)
- Low-power instrumentation amplifier INA118P (Texas Instruments)

Power monitor

- Photon microcontroller (Particle)
- ZigBee wireless communications module XB24-AUI-001 (Digi International)
- 400 mV/A Hall effect sensor ACS723 (Allegro)
- 132 mV/A Hall effect sensor ACS717 (Allegro)
- 5 V switch-mode power supply ECE05US05 (XP Power)

Door sensor

- Photon microcontroller (Particle)
- ZigBee wireless communications module XB24-AUI-001 (Digi International)
- 2.2 Ah lithium thionyl chloride primary cell LS 14500W (Saft)
- 5 V voltage regulator MCP1700 (Microchip)

Fall detector

- FRDM-KLZ25 M0+ MCU (NXP Semiconductor)
- ZigBee wireless communications module XB24-AUI-001 (Digi International)
- I2C 12-bit digital tri-axis accelerometer MMA8452Q (NXP Semiconductor)
- I2C barometric pressure sensor BMP180 (Bosch)
- 40 mAh lithium-ion polymer battery PPPRT-11316 (SparkFun)
- 3.3 V 400 mA linear voltage regulator LM2937 (Texas Instruments)

On-person device

- FRDM-KLZ25 M0+ MCU (NXP Semiconductor)
- UART Global System for Mobile Communications module GL865 DUAL (Telit)
- Quad-band cellular duck antenna GSM850 (S. P. K. Electronics)
- Automatic gain control microphone amplifier MAX9814 (Maxim Integrated)
- 0.5 W speaker PPCOM-09151 (SparkFun)
- UART Global Positioning System module EM-506 (USGlobalSat)
- 1000 mAh 3.7 V lithium-ion polymer battery PPKIT4652 (SparkFun)
